# Auditory imagery ability influences accuracy when singing with altered auditory feedback

**DOI:** 10.1177/10298649231223077

**Published:** 2024-02-15

**Authors:** Courtney N. Reed, Marcus Pearce, Andrew McPherson

**Affiliations:** Loughborough University London, UK; Queen Mary University of London, UK; Queen Mary University of London, UK; Imperial College London, UK; Queen Mary University of London, UK

**Keywords:** musical imagery, auditory perception, singing, performance, musical training

## Abstract

In this preliminary study, we explored the relationship between auditory imagery ability and the maintenance of tonal and temporal accuracy when singing and audiating with altered auditory feedback (AAF). Actively performing participants sang and audiated (sang mentally but not aloud) a self-selected piece in AAF conditions, including upward pitch-shifts and delayed auditory feedback (DAF), and with speech distraction. Participants with higher self-reported scores on the Bucknell Auditory Imagery Scale (BAIS) produced a tonal reference that was less disrupted by pitch shifts and speech distraction than musicians with lower scores. However, there was no observed effect of BAIS score on temporal deviation when singing with DAF. Auditory imagery ability was not related to the experience of having studied music theory formally, but was significantly related to the experience of performing. The significant effect of auditory imagery ability on tonal reference deviation remained even after partialling out the effect of experience of performing. The results indicate that auditory imagery ability plays a key role in maintaining an internal tonal center during singing but has at most a weak effect on temporal consistency. In this article, we outline future directions in understanding the multifaceted role of auditory imagery ability in singers’ accuracy and expression.

Imagery allows us to perceive or anticipate details of an event or action without it occurring. Imagined representations play a preparatory role in motor control and share neural and behavioral similarities with executed actions (Cumming & Williams, 2012; Halpern & Zatorre, 1999; Kleber et al., 2007; Kosslyn et al., 2011; Trusheim, 1991; Zatorre et al., 2007). Musical imagery encompasses all sensory aspects of a musical experience without or prior to action or sound production (Godøy & Jørgensen, 2001; Keller, 2012; Trusheim, 1991). Existing research focuses largely on auditory imagery, the ability to consciously imagine the qualities of a sound. Auditory imagery is used to prepare the body and instrument to properly execute and adapt technique in performance. Personal experiences (e.g., instruction, practice, time spent in performing environments) form imagery associations, which are therefore highly individual. Imagery use will also vary based on specific background and training (Bianco et al., 2018; Fontana et al., 2015; Pfordresher, 2019).

Musicians notably use auditory imagery in *audiation*. Audiation can be thought of as the musical equivalent of thought in linguistic communication (E. E. Gordon, 1999). It is how we construct ideas and understanding and give meaning to what we hear (E. E. Gordon, 2011). Musicians commonly use the term to mean hearing music covertly in the mind, using mental imagery, without overt playing or singing (Brodsky et al., 2008; Halpern & Overy, 2019; Pfordresher & Halpern, 2013). This silent rehearsal is useful while exploring technical and expressive aspects (Bailes, 2006; Cumming & Williams, 2012; Holmes, 2005; Loimusalo & Huovinen, 2016) and for sensorimotor coordination (Fontana et al., 2015; Keller, 2012; Leman & Maes, 2015; Pfordresher, 2019) in performance.

## Adapting to altered auditory feedback

Overt auditory feedback in real-world settings is not always ideal; altered auditory feedback (AAF) in live performance environments can happen through masking, noise, speech distractions, improper monitoring, and uncontrolled resonances from the space itself. These conditions can be managed by recalling auditory imagery to monitor performance and anticipate action-sound consequences. Action-sound outcomes are ideally formed in rehearsal for improved accuracy in performance (Goebl & Palmer, 2008; MacRitchie & Milne, 2017). Studies with pianists found that auditory imagery developed in rehearsals allowed musicians to recall and play accurately with reduced or even no auditory feedback (Brown & Palmer, 2012; Edmonson, 1972; Finney & Palmer, 2003; Highben & Palmer, 2003). Understanding action-sound outcomes through auditory imagery extends to the use of articulation, dynamics, and expressivity (Bishop et al., 2013). Auditory imagery also assists with motor coordination and expression in duet and group performances, in adapting to other players and communicating through expression and gesture (Brown & Palmer, 2012; Davidson, 2012; Highben & Palmer, 2003; Zatorre et al., 2007).

## Measuring imagery ability

The Bucknell Auditory Imagery Scale (BAIS) is used to determine the vividness (BAIS-V) and control (BAIS-C) of auditory images (Halpern, 2015). The questionnaire uses Likert-type scales for self-assessing musical, environmental, and spoken-voice sound sources. BAIS self-reports have been found to correlate significantly with behavioral aspects of musicality. Higher scores on BAIS-V correlated with better pitch imitation (Pfordresher & Halpern, 2013), recall and recognition of transposed, reversed, and serially shifted melodies (Greenspon et al., 2017), and the occurrence of involuntary musical imagery, or earworms (Floridou et al., 2014). Higher scores on BAIS-C correlated with better prediction of melodic movement (Gelding et al., 2015) and anticipation of tempo changes (Halpern, 2015).

BAIS is also associated with aspects of neural musical processing. Neural activation was found to be greater in participants with higher BAIS-V scores in the right anterior superior temporal gyrus (secondary auditory cortex) and right dorsolateral prefrontal cortex (involved in working memory) during encoding of imagined melodies (Herholz et al., 2012). Higher average BAIS score correlated with activity in the right secondary auditory cortex and right intraparietal sulcus in mental reversal of a melody (Zatorre et al., 2010). BAIS-V scores also correlated with gray matter volume in the left inferior parietal lobule and left supplementary motor area (Lima et al., 2015). These areas have also been implicated in functional studies of musical imagery (Foster et al., 2013; Halpern & Zatorre, 1999; Zatorre et al., 2010).

## Singing and auditory imagery

The voice provides a unique case for studying auditory imagery, as singing does not provide the same external feedback as other instruments; for instance, a pianist can rely somewhat on one-to-one key mappings. Auditory imagery is essential for translating ideal sound to physiological expression (Clark et al., 2011); without direct tactile connections (Hemsley, 1998; Hines, 1983), vocalists depend on internal kinesthetic feedback (Larson et al., 2008) and understanding of action-sound outcomes through auditory imagery (Salaman, 1989). Previous work has demonstrated that singers may be more susceptible to pitch-shifts than keyboard players due to perceived response–effect associations and the coordination that singers learn in normal circumstances (Pfordresher & Mantell, 2012). When instructed to ignore or adjust to pitch-shifted feedback, non-musicians employed the left supramarginal gyrus and primary motor cortex or the dorsal premotor cortex, respectively, which are involved in sensorimotor interaction (Zarate & Zatorre, 2008). Trained singers, by contrast, exhibited neural activation in bilateral auditory areas and left putamen; when instructed to adjust to the feedback, they recruited the anterior cingulate cortex (ACC), superior temporal sulcus, and putamen. This study demonstrated how, using the sensorimotor foundations observed in non-musicians, singers learn to monitor their pitch and vocal-motor programs with pitch-shifted feedback to achieve their desired vocal output (Zarate & Zatorre, 2008).

## The present study

In the present study, we asked how effectively skilled singers with different auditory imagery abilities adapt to non-ideal performance conditions and different types of AAF. We aimed to determine how consistently participants would maintain their intonation and timing when they relied on auditory imagery rather than expected auditory feedback. We used temporal delays and pitch-shifted AAF and forced participants to rely on imagery in several tasks in which they audiated portions of a song while performing. We thus examined how varying performance conditions influenced singing accuracy in comparison with non-altered conditions. We hypothesized, first, that greater auditory imagery abilities, self-reported using BAIS, would correlate with greater accuracy in the presence of AAF, consistent with previous studies (Pfordresher et al., 2015). In previous studies, BAIS score was found to be only weakly positively correlated with years of musical training (Halpern, 2015; Herholz et al., 2012; Pfordresher & Halpern, 2013). We therefore hypothesized, second, that musicians with more experience of performing, rather than more years of studying music theory formally, would have greater auditory imagery ability as the result of performing in a variety of settings and circumstances, and would therefore perform better with AAF.

## Method

### Participants

In all, 16 musicians (7 male, 9 female), aged 22–37 years (*M* = 28), were recruited through an open call online and via email lists for musical groups in London, UK. Participants needed to be musically active and “be able to sing confidently and do so with reasonable pitch accuracy” in unaccompanied performance. Demographic and experience data were collected at sign-up (Supplemental Table 1). Participants represented a variety of nationalities, were all fluent English speakers, and lived in the United Kingdom at the time of the study. Nine participants were primarily vocalists, six with formal voice training outside compulsory schooling. The remaining seven included two pianists, two guitarists, one flutist, one dhol player, and one electronic digital instrumentalist; six had formal training on their instrument (Supplemental Table 1).

Participants provided written informed consent for collection, inclusion, and publication of their data, including anonymized questionnaire results and audiovisual recordings. Participants were paid for their time following the completion of the study. The study was reviewed and approved by the Queen Mary University of London Ethics of Research Committee under QMREC2125.

### Materials

Imagery has strong emotional associations (Zagacki et al., 1992), so we believed that participant-chosen songs would provide stronger imagery, while being enjoyable and avoiding significant time costs and anxiety as confounding factors associated with learning or sight-singing an unfamiliar piece. Participants therefore brought “a solo piece/excerpt that [they] enjoy singing and can perform accurately without accompaniment,” 2–3 min in length, in any style. Song choices were agreed beforehand; the key and tempo were decided at the start of the study (Supplemental Table 2) to ensure comfort and a consistent reference between repetitions. Participants completed the Goldsmiths Music Sophistication Index (Gold-MSI; Müllensiefen et al., 2014) to assess musical background and demographics and the BAIS to assess auditory imagery ability.

### Apparatus

**Figure 1.**
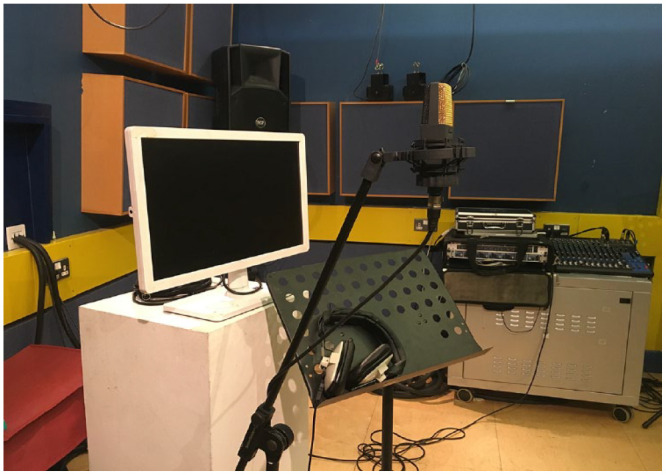
The recording and monitoring setup used for completing the tasks and receiving visual cues for audiation.

The study was conducted in an isolated, acoustically treated recording room at Queen Mary University of London (Figure 1). Directions and auditory stimuli were given via a pair of RCF ART 412-A speakers or with a Beyerdynamic DT100 closed, circumaural studio headset. The headphones offer ~20 dBA of noise attenuation and were chosen to provide isolation for AAF stimuli from the unaltered voice. Participants were recorded with an AKG C414B-XLII condenser microphone, via a Yamaha MG16XU mixing console and Universal Audio 4-710d Tone-Blending mic preamp, into Logic Pro X running Mac OS 10.14.1 in a neighboring mixing studio. Visual stimuli created in MAX/MSP (Cycling’74) were presented on a 24-in. BenQ LCD monitor approximately 2 in. away.

### Tasks

The study involved three tasks, each conducted using six AAF conditions. The tasks were performed in the following order:

1. Normal. Sing the piece as written.
2. Toggled. Alternate between singing aloud and audiating as visually instructed.
3. Toggled and Voice Distraction (TVD). As per the Toggled task, with an additional stimulus of external dialogue during the audiated sections.

**Figure 2.**
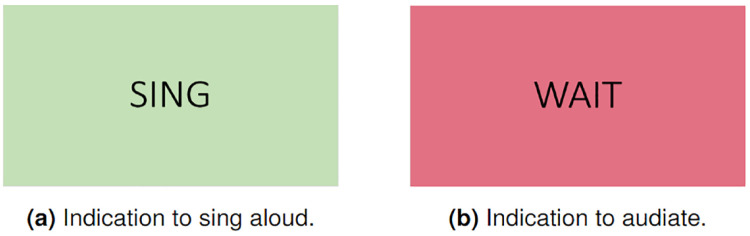
Visual stimuli displayed on the monitor during the Toggle and Toggled & Voice Distraction tasks.

In Toggled and TVD tasks, participants were instructed to either “sing” aloud or “wait,” while audiating, indicated by the monitor (Figure 2). The screen alternated on a random interval of 5–15 s; this toggling was designed to force audiation and reliance on musical imagery. In the TVD task, a podcast conversation (Fermat’s Last Theorem, *In Our Time*, Melvin Bragg, BBC Radio 4, 25 October 2012) was also played in audiated sections. Audible speech is found consistently to be more disruptive to tasks, including audiation in reading, than other sound distractions (Vasilev et al., 2018; Venetjoki et al., 2006).

#### Auditory feedback conditions

Each task included six feedback conditions (2 control, 2 DAF, 2 upward pitch-shifts, 18 total performances):

- Normal feedback (NF) (control, room feedback).
- Headphone feedback (HF) (control, setup latency).
- 200-ms delay.
- 600-ms delay.
- +quarter-tone pitch-shift.
- +whole-tone pitch-shift

Research in the field of speech, language, and hearing has shown a delay of 200 ms to be the most disruptive to speech production (Atkinson, 1953; Black, 1951; Fairbanks, 1955; Sasisekaran, 2012; Zimmermann et al., 1988), functionally interrupting the action-effect path in sensorimotor coordination (Howell, 2004; Howell et al., 1983). The 600-ms delay has qualitatively different effects; longer delays inhibited monitoring of vocal parameters and internal beat subdivisions (Bartlette et al., 2006; Finney & Warren, 2002; Lee, 1950; Pfordresher & Palmer, 2002; Zarate & Zatorre, 2008) and potentially disrupted longer phrases (Howell et al., 1983). We used upward pitch shifts because most singers drift downward over time when they sing on their own (Howard, 2007; Mauch et al., 2014), and we wanted to counteract this tendency, avoiding the risk of exaggerating it and confounding our results. The narrower quarter-tone pitch shift was intended to provide a sense of chorusing and the wider whole-tone shift a distinctly out-of-key sensation, requiring participants to ignore it.

AAF stimuli were provided via the headphones with added gain to mask the unaltered voice (Malloy et al., 2022). Gain was set to a level that was “comfortable, but you should not be able to hear your voice outside of the headphones.” Ultimately, this was ~80–82 dB SPL. This would not mask bone conduction of the unaltered voice, which should be noted, but minimizes veridical feedback. DAF was introduced with Logic Pro X’s built-in Sample Delay plug-in. Pitch shifting was added via the built-in Pitch Shifter plug-in, set with 0.0-ms delay, Latency Comp, and the smallest I/O buffer size (32 samples) for latency reduction. Participants listened to direct monitoring on the track using this plug-in. Using an oscilloscope, the round-trip latency (RTL) for this monitoring was measured to be approximately 7 ms, and so would not provide noticeable latency beyond the DAF. Raw vocal audio was recorded on a separate track and bounced in place to ensure no latency was introduced in files used for analysis.

### Procedure

The NF condition was always performed first as a control for the HF condition and to familiarize participants with each task before adding AAF demands. All participants confidently sang their piece unaccompanied in the NF condition, without additional stimuli (assessed qualitatively by Reed, the first author, a semi-professional singer), and were able to proceed. The remaining conditions were presented in a randomized order.^1^ An ideal performance would involve maintaining a consistent tonal center and tempo throughout the piece, effectively ignoring AAF and singing as in the NF control condition. With pitch-shifted conditions, the internal tonal center should be maintained and, while performing under DAF, a continuous and consistent pulse. In Toggled and TVD tasks, audiating should not influence tempo and key, nor cause late or early entries, “as if someone has just muted you for a short time.” Screen changes were shown beforehand, and participants also spoke into the mic to hear the AAF before each condition, to reduce surprise. Participants were then instructed before each performance to

sing the piece as you did during the first run-through, ignoring any auditory feedback you hear in the headphones. Sing the piece as you would normally, keeping in key and staying in time. If you make a mistake, keep going; if you find you are off-key or are changing tempo, stay consistent and continue to the end of the piece with your new key or new tempo. It is important to not stop and to continue on singing as well as you can.

For each repetition, the starting pitch and first two bars’ melody were provided via Logic Pro X on the default Steinway Grand Piano software instrument patch. A reference tempo count-in was provided with Logic’s digital metronome. The full study took 1 hr for each participant including time spent completing questionnaires and approximately 45 min of singing.

### Data processing

Each performance was processed in Sonic Visualizer (Cannam et al., 2010) and Tony (Mauch et al., 2015). Sung pitches were extracted using Tony’s pYIN algorithm (Mauch & Dixon, 2014; Mauch et al., 2014) and corrected where necessary. Note onsets and beats, based on the score of the piece that was sung, were annotated manually in Sonic Visualizer. Participants were not expected to be able to ignore AAF perfectly; therefore, we defined accuracy as the extent of deviation from expected tuning and timing quantified objectively, rather than in terms of expressivity or esthetics.

Kennedy and Kennedy’s (2007) definition of intonation as “the act of singing or playing in tune” (p. 235) requires an existing tonal reference; with unaccompanied singers, the reference is internal (Mauch et al., 2014) and cannot be measured directly. Therefore, tonal reference deviations (TRDs), measured in semitones, proposed by Dai et al. (2015) were calculated for estimated intonation. Temporal drift over time is less well understood but is found to be generally inconsistent, and the degree of drift varies between individuals (Repp, 2002). Central clock variance found in isochronic tapping with different limbs suggests that musical experience or inclination, among other factors, influences natural drift tendency (Collier & Ogden, 2004). Human beings use contextual information for timing, which suggests that variation should be examined across a series of onsets (Madison, 2004). In previous study of DAF in musical performance, the coefficient of variation (CV) was used to measure timing variability (Pfordresher & Palmer, 2002). We used this measure to address temporal drift. We were unable to determine timing in audiated sections; we calculated the absolute average number of missed beats (MBs), adjusted for the length of the audiated section, to represent internal temporal reference. We therefore used TRD, CV, and MBs as measures of accuracy.

#### TRD

A pitch track of expected notes was created from the score and aligned to sung notes (in Toggled and TVD tasks, audiated pitches were excluded). Sung and expected pitches were converted to musical pitch in semitones (Dai et al., 2015). TRD estimates a tonal reference trajectory over time with score normalization, adjusting sung pitches to expected pitches (Dai et al., 2015). This estimate approximates the local, internal reference, with respect to neighboring pitches. Because the internal reference is based on pitch memory (Mauch et al., 2014), a sliding window can be used to estimate the magnitude of its trajectory over time. We used a size *N* = 5 window, which represents natural deviation by judging each note against the two notes directly before and after (five notes in total). The standard deviation of this tonal reference curve provides a measure of TRD, representing internal reference fluctuation (for further information, see Dai et al., 2015). As this measure estimates the reference pitch, small errors in tuning would not be penalized as much as wholly inaccurate intervals. With the sliding window, if a participant abruptly loses their key, TRD penalizes the initial error. It is subsequently smoothed by the overall trajectory of the deviation if the participant continues consistently. We expected participants with better auditory imagery to have lower TRD.

#### CV

CV is calculated as the standard deviation of the inter-onset interval (IOI) divided by the mean IOI. The duration between each note and its predecessor (omitting the first beat of a performance) is calculated in milliseconds as an IOI. For Toggled and TVD tasks, the beat before the visual change is marked and audiated sections are assumed to continue at the same tempo from the last beat vocalized. Inconsistencies in musical timing at a local level are normal, especially when performing solo; without the reference of other players, this drift would be less noticeable; CV represents dispersion around the mean tempo and depicts the general deviation. As in the case of TRD, if a participant changed to a new tempo, the deviation would be penalized but not compounded if the participant continued consistently.

#### MBs: Drift during audiation

We used MBs in audiation-inclusive tasks. Audiated tempo is assumed the same beats per minute (BPM) as the last beat sung. The number and length of audiated sections varied on the random toggle and the duration of the piece. Therefore, MBs were averaged according to the length and frequency of the audiated sections. We anticipated that greater imagery ability would produce more accurate timing, consistent beat keeping, and fewer MBs while audiating.

### Data analyses

We conducted two analyses using different baselines. First, participants’ AAF-condition performances were examined against their individual control-condition performances to determine the effect of BAIS on consistency between AAF and control-condition performances. This also accounted for individual differences between control-condition performances and the performance of different participant-chosen pieces. In each task, a participant’s AAF-condition scores were normalized with respect to their control-condition score. This individual-adjusted score represented how well the participant performed with AAF in comparison with their control-condition performance. We used a difference score for each measure of accuracy, subtracting baseline accuracy from the accuracy of each AAF-condition performance. Thus, an adjusted score of 0 indicated the same error as the control condition (consistent performance), a positive score more error, and a negative score less error.

Second, a group-adjusted analysis was performed. This accounted for some participants having more error than others in their control-condition performances. Again, we used a difference score for each measure of accuracy, subtracting the group average representing baseline accuracy, to normalize AAF-condition performance scores to the average control-condition score of all the participants who completed each task (Table 1). The group-adjusted score thus represented the extent to which the participant’s performance with AAF was better or worse than the average control performance.

**Table 1.** Group-averaged accuracy measures for adjustments.

Task	Group accuracy		
	TRD	CV	MBs
Normal	0.47	9.74	N/A
Toggled	0.46	9.09	0.82
Toggled & Voice Distraction	0.43	8.99	1.10

TRD: tonal reference deviation; CV: coefficient of variation.

We conducted 2 × 3 × 4 mixed analyses of variance (ANOVAs) to compare performances with respect to each measure of accuracy. All the participants had at least a year’s experience of performing before taking part in the study (*M* = 10.9 years) and had studied music theory formally (*M* = 6.6 years). Participants’ BAIS-V scores ranged from 4.14 to 6.57 (*M* = 5.19, *SD* = 0.7) and BAIS-C ranged from 3.79 to 6.43 (*M* = 5.23, *SD* = 0.8). There were no statistically significant differences between BAIS scores with respect to primary instrument or years of formal training on that instrument, and BAIS subscales were positively correlated (Supplemental Result SR-1). We therefore used an average BAIS score to group participants for this ANOVA. We used a median split (*M* = 5.02) to categorize participants into two groups of high-BAIS and low-BAIS scorers. Thus BAIS group was the between-subjects independent variable, the repeated-measures (within-subjects) independent variables were task (Normal, Toggled, and TVD) and condition (NF, HF, 200 and 600 ms delay, and + quarter-tone and + whole-tone pitch-shift), and the dependent variables used in separate ANOVAs were each measure of accuracy (TRD, CV, and MB).

## Results

Full-factorial results can be found in the Supplemental Results (SR). Participant demographics by BAIS group are presented in Supplemental Table 4. First, we asked if the self-selected pieces introduced complexity covariates and found none (SR-2). AAF conditions were delivered via headphones, so we compared NF and HF tasks and found no significant differences between them. We did not find a link between BAIS score and accuracy in the control-condition tasks (SR-3), so we used the HF task as a control condition for the other AAF conditions, assuming no effect of imagery abilities on accuracy with unaltered HF.

### Tonal deviation

**Figure 3.**
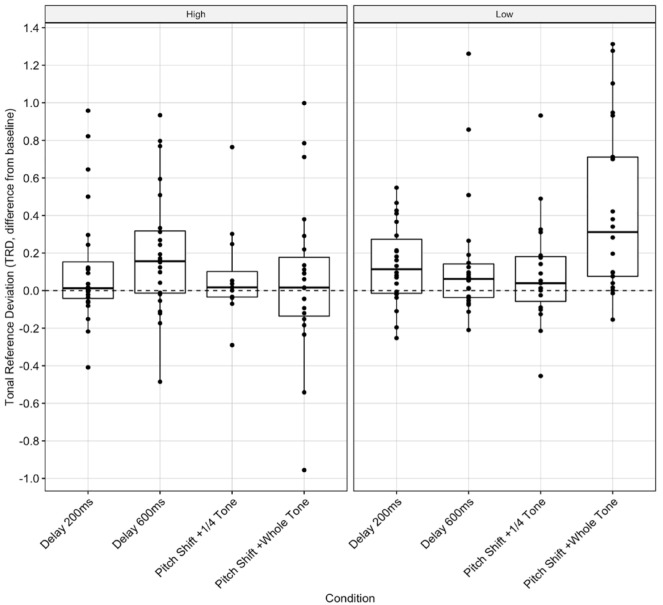
Tonal deviation: Individual-adjusted TRD (semitones) score for the AAF conditions, by BAIS group. NB: In the box plots, each dot represents a participant, demonstrating the distribution of the performances. The horizontal line reflects the median performance.

For individual-adjusted TRD, we found a significant two-way interaction between BAIS group and task, *F*(2, 144) = 3.304, *p* = .040, and BAIS group and condition, *F*(3, 144) = 3.628, *p* = .015. According to Bonferroni-adjusted pairwise comparisons, there were significant differences between groups in the whole-tone pitch-shift condition, *t*(162) = –2.97, *p* < .001 (Figure 3, SR-4), with high-BAIS participants having lower TRD (*M* = 0.08, *SD* = 0.45 semitones) than low-BAIS participants (*M* = 0.46, *SD* = 0.47 semitones).

**Figure 4.**
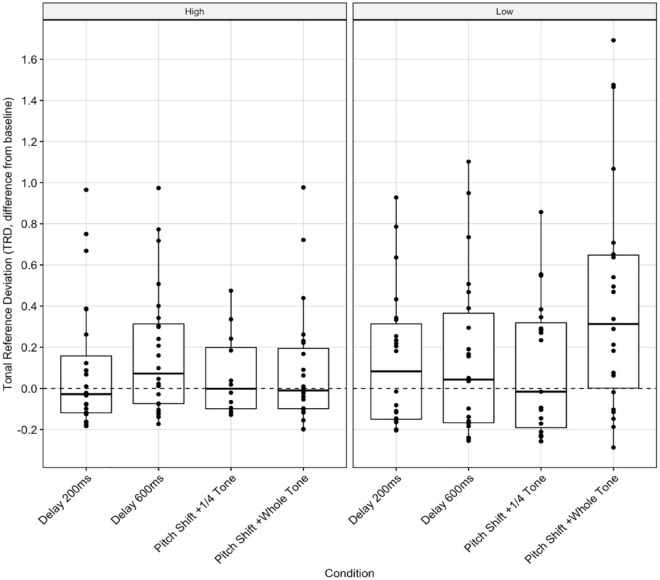
Tonal deviation: Group-adjusted TRD score (semitones) for the four AAF conditions, by BAIS group.

For group-adjusted TRD, we found no significant interactions between the independent variables. Again, Bonferroni-adjusted pairwise comparisons indicated significant differences between groups in the whole-tone pitch-shift condition, *t*(160) = –2.00, *p* = .047 (Figure 4, SR-5), with high-BAIS participants producing less TRD (*M* = 0.13, *SD* = 0.3 semitones) than low-BAIS participants (*M* = 0.43, 0.57 semitones).

#### Temporal deviation

**Figure 5.**
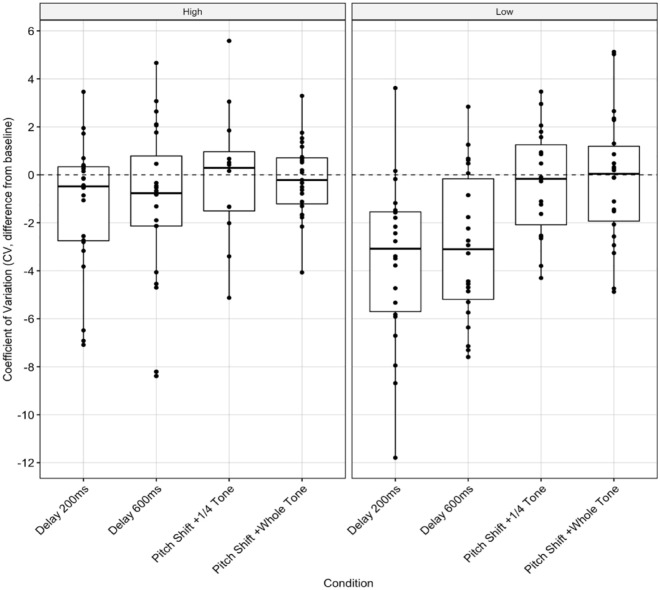
Temporal deviation: Individual-adjusted CV score for the four AAF conditions by BAIS group.

There were significant main effects of condition, *F*(3, 144) = 7.321, *p* < .001, and BAIS group on individual-adjusted CV, *F*(1, 144) = 7.323, *p* = .008, but no significant interactions between them. Bonferroni-adjusted pairwise comparisons indicated significant differences between groups for both 200 ms DAF, *t*(160) = 2.85, *p* = .005, and 600 ms DAF, *t*(160) = 2.34, *p* = .021. While high-BAIS participants performed consistently in DAF (200 ms: *M* = -1.35, *SD* = 2.82; 600 ms: *M* = −1.04, *SD* = 3.43) and control tasks, some low-BAIS participants had lower timing error (200 ms: *M* = −3.67, *SD* = 3.44; 600 ms: *M* = −2.97, *SD* = 3.08) in DAF tasks (see negative adjusted CV scores, Figure 5, SR-6).

**Figure 6.**
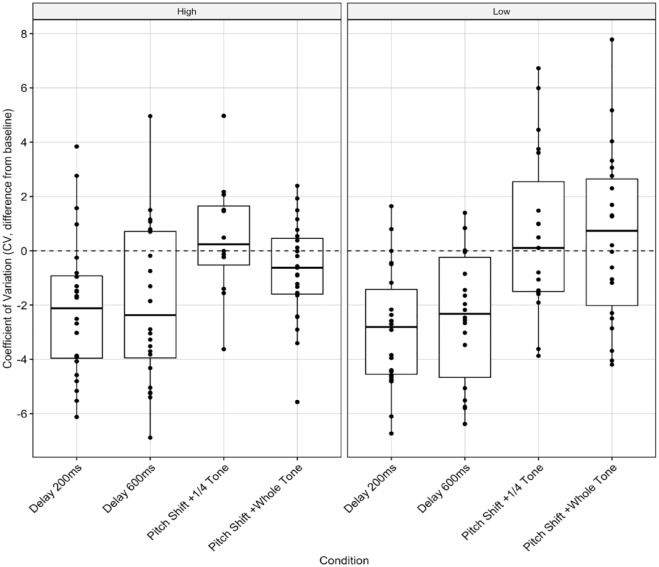
Temporal deviation: Group-adjusted CV score for the four AAF conditions, by BAIS group.

We found a significant main effect of condition on group-adjusted CV, *F*(3, 144) = 12.721, *p* < .0001, but no significant interactions between them. When factoring in the group average, high-BAIS (200 ms: *M* = −2.07, *SD* = 2.63; 600 ms: *M* = −1.99, *SD* = 2.95) and low-BAIS participants (200 ms: *M* = −2.93, *SD* = 2.27; 600 ms: *M* = −2.58, *SD* = 2.32) had similar adjusted CVs. Both groups produced slightly better than average performances with DAF, mirroring the individual-adjusted analysis (Figure 6, SR-7).

For Toggled and TVD tasks, we found no significant main effects or interactions for either individual-adjusted (SR-8) or group-adjusted MBs (SR-9).

#### Musical experience

Follow-up analyses examined links between participants’ musical experience and BAIS score (Supplemental Table 4). The performance experience of the high-BAIS group ranged from 9 to 24 years (*M* = 15.13, *SD* = 5.25), while the performance experience of the low-BAIS group ranged from 1 to 12 years (*M* = 6.63, *SD* = 4.14). The years of formal theory study of the high-BAIS group ranged from 1 to 20 years (*M* = 9.5, *SD* = 6.21), and those of the low-BAIS group from 0.5 to10 years (*M* = 4.87, *SD* = 4.35). There was a weak, nonsignificant correlation between participants’ years of studying music theory formally and BAIS score (*p* = .22), and no significant difference between the two BAIS groups’ years of studying music theory, indicated by a two-sample *t*-test (*p* = .1). However, there was a strong correlation between participants’ years of experience of performing and BAIS score, *r* = .6, *F*(1, 14) = 7.81, *p* = .014. Two-sample *t*-tests indicated that BAIS groups’ years of experience of performing also differed significantly, *t*(13) = −3.6, *p* = .002.

The results of full and partial correlation analyses showed that these differences were attributable to the effects of the whole-tone pitch-shift condition on TRD, and of the 200 ms DAF condition on CV. There was a moderate negative correlation between TRD and BAIS score, *r*(43) = −.44, *p* = .003. The partial correlation between BAIS score and per-participant TRD when controlling for years of experience of performing in the whole-tone pitch-shift condition remained significant, *r* = −.45, *F*(1, 45) = −3.25, *p* = .002. We found a non-significant correlation between TRD and years of experience of performing. The partial correlation, when controlling for BAIS, remained nonsignificant. This supports the effect originally observed: higher BAIS score correlates with less TRD with pitch shifts, even when controlling for experience of performing. The relationship between TRD and years of experience of performing is fully explained by differences in participants’ auditory imagery abilities assessed in the partial correlation analysis.

We found non-significant correlations between CV, BAIS score, and years of experience of performing (all *p* > .053). When we controlled for the latter, the partial correlation between BAIS score and CV in the 200 ms DAF condition remained non-significant (*p* = .06). When we controlled for BAIS score, the correlation between years of experience of performing and CV also remained non-significant (*p* = .07). This too supports the effects originally observed: while there were significant effects of condition on CV, these were unaffected by BAIS score.

## Discussion

We hypothesized that greater auditory imagery abilities, self-reported using BAIS, would correlate with greater tonal accuracy when singers were presented with pitch-shifted feedback and greater temporal accuracy with delayed auditory feedback. We also hypothesized that participants with more years of experience of performing, as opposed to years of studying music theory formally, would have greater auditory imagery ability, and therefore perform better with AAF. We found that higher BAIS score was linked to greater tonal accuracy but not temporal accuracy in DAF. We also found that BAIS score was linked to years of experience of performing but not years of studying music theory.

There were significant interactions between BAIS group and both task and condition on individual-adjusted TRD. The whole-tone pitch-shift condition (Figure 3) and the TVD task (SR-4) had significantly greater effects on participants with low BAIS scores, resulting in higher TRD. Singers with high BAIS scores appeared better able to maintain tonal accuracy than in their control-condition performances. There was a significant difference between BAIS groups’ individual-adjusted TRD when performing the TVD task. This suggests that auditory imagery may help in maintaining a tonal reference while audiating. Group-adjusted analysis (Figure 5) indicated a main effect of condition but not BAIS. Previous work has shown large effects of BAIS in poor-pitch singers (Greenspon et al., 2017; Pfordresher & Halpern, 2013; Pfordresher et al., 2015); there appears to be no significant effect of BAIS score on tonal accuracy when comparing different musicians, as seen here. This suggests that auditory imagery does not provide individuals with the ability to outperform others with similar imagery skill but helps them perform consistently with AAF.

The significant difference between the groups’ performance on the whole-tone pitch-shifted task indicates that this type of AAF is more disruptive to low-BAIS singers, given that about half of our participants had ⩾0.4 semitones TRD. Similar deviations are common when singing with others, for instance, in unaccompanied choral settings. Auditory feedback is essential for staying in tune and blending with other voices (Howard, 2007), but singers with lower BAIS scores may find it harder to sing in tune. However, we did not find this difference when the shift was only a quarter-tone, suggesting that there is a threshold above which it is harder to sing in tune with AAF. Research in the field of speech, language, and hearing has shown listeners are less able to compensate, using opposition or matching strategies, in the presence of larger pitch shifts (Daliri et al., 2020; MacDonald et al., 2010); smaller shifts might feel more like chorusing (one participant described it as a “robot filter”), in which a singer expects to be able to make use of the feedback from what they can hear around them. The whole-tone shift might be large enough to disrupt singers’ ability to compensate. In the present study we did not examine how the direction of the pitch shift influenced drift; it would be worth exploring this empirically because unaccompanied singers typically drift downward.

Franken et al. (2018) argue that listeners compensate for pitch-shifting because their motor system attempts to minimize the difference between predicted and perceived feedback, and they use opposition and/or matching depending on whether they think the discrepancy is internal or external. In the present study, high-BAIS participants might have relied more on internal predictions (Jones & Keough, 2008; Zarate & Zatorre, 2008). Franken et al. (2023) found that singers who adjusted as though the discrepancy were internal had previously rated themselves as less musical. This would be consistent with our finding that low-BAIS participants reported fewer years of experience of performing.

We found less straightforward relationships between temporal deviation and BAIS score. There were significant effects of BAIS group and condition on individual-adjusted CV, but no interaction between them. In the low-BAIS group, there were significant effects of the 200-ms DAF and 600-ms DAF conditions, although low-BAIS participants had lower CV in these conditions than in the control condition (Figure 4), which cannot be attributed to BAIS score as there was no interaction between group and condition. Similarly, there was a significant effect of condition in the group-adjusted analysis but no effect of group (Figure 6). Both groups performed slightly better in both DAF conditions than the control condition.

The effects on speech of frequency- (i.e., pitch-) altered feedback and DAF are different. In the presence of pitch-shifted feedback, people change their speech patterns to match expectations better; in the presence of DAF, they may produce onset disfluencies (Burnett et al., 1998; Purcell & Munhall, 2006) or stutters where “the same onset consonant is produced 2 or more times without a clear intervening vowel” (Malloy et al., 2022, Table 1, p. 6). This would explain why we found a link between BAIS score and pitch-shifted feedback but not DAF, which requires not only auditory imagery but also the use of motor behavior. Longer delays are particularly difficult to deal with as listeners are more aware of the mismatch between what they are expecting and what they are hearing; in short, they are both neurologically and cognitively disruptive (Malloy et al., 2022).

Our results may also arise from the fact that BAIS does not measure temporality, required for singing with delays. Internal pulse is reliant on both auditory and kinesthetic imagery, as auditory-motor interaction utilizes the predictive role of the motor system to judge timing (Cannon & Patel, 2021; Proksch et al., 2020), even without movement (C. L. Gordon et al., 2018). Sensorimotor synchronization (SMS) ability positively correlates with auditory imagery ability and years of playing musical instruments (Pecenka & Keller, 2009). Auditory imagery also enables individuals to map planned motor images accurately, including note timing, mental tempo representations (Pfordresher & Palmer, 2002), and expressive SMS (Colley et al., 2018). These abilities may vary according to whether individuals prefer to make use of somatosensory or auditory feedback (Lametti et al., 2012), or which subsystem of the vocal mechanism—involving articulatory or laryngeal control—is more active (Weerathunge et al., 2022). It may be better to use the Multi-Modal Imagery Association (MMIA) model, which links auditory and kinesthetic imagery through sensorimotor associations (Pfordresher et al., 2015), to investigate the complex timing systems used in singing.

It may be that our participants performed better with DAF because they introduced other, external, elements of timekeeping, such as foot tapping or body sway. Even non-musical individuals have been found to have accurate absolute tempo references preserved in long-term memory (Levitin & Cook, 1996); absolute tempo is associated with tactus, while internal tempo is associated with rhythmic period representations or body-based references (Gratton et al., 2016). Urges to move to music are similarly driven by an internal representation of temporal regularity (Hosken, 2018; Senn et al., 2019; Vuust et al., 2018). Visual stimuli associated with timekeeping have been found to help people cope with DAF in speech (Malloy et al., 2022); some participants in the present study conducted beat patterns in front of their bodies, thus employing kinesthetic-visual references.

Participants’ goals for performance are also likely to have been different in the different AAF conditions. The control conditions represented typical solo singing, which usually prioritizes expression (Müller et al., 2010) and relies on salient perceptual onsets rather than strict metronomical timing (Coath et al., 2009). Human beings are thought to be naturally lax when determining isochrony; we learn to perceive repetition and timing through our experience of natural stimuli and internal periodicity, which are rarely precise, and therefore perceive regularity even when some drift is present (Madison, 2004; Madison & Merker, 2002). Compared with human perception of timing, CV is strict. Our participants may have been more focused on timing in the DAF than the control conditions. To give one example, the behavior of Participant 9 (P9) and their background as a performer may suggest why we observed less variation in the DAF conditions. P9’s CV in the control task was 15.43 but was only 3.64 in the 200 ms-DAF task (i.e., an individual-adjusted score of −11.79, the lowest of all such scores). Their timing in the control task was very free; their chosen song had frequent syncopations, and P9 placed emphasis on, and thereby lengthened, certain words in the text. P9’s approach to the DAF task was quite different. They danced as they sang, using full-body sway, arm movements, and foot tapping. They used glottal stops to articulate the beats audibly within longer-held notes. They described their enjoyment of meeting the challenge, saying they had focused on carrying out the task, not on recreating their initial performance. Given that P9 is an experienced experimental and electro-pop performer, it is likely that they had had previous experience of DAF.

There were no effects of BAIS group, task, or condition on the number of MBs in audiated sections, which further supports the limited effect of BAIS score on temporal deviation. Rhythmic stability appears to be mostly unaffected when the individual is switching between audiating and singing. The greatest drift was about ±2 beats compared with control tasks. Most participants averaged <1 MB in any audiated section. This suggests awareness of the current tempo and ability to adjust, as participants did not default back to a remembered tempo once they began audiating, but rather continued where they left off. One participant who struggled with DAF said, “I could feel I was going too slow, but it became so hard to stop.” Other comments also addressed tactile experiences; similar effects have been reported in the speech, language, and hearing research literature with reduced speaking rate as a compensation for DAF (Fairbanks, 1955; Finney & Warren, 2002). It is likely that DAF disrupts the neural and sensorimotor feedback loops active when individuals monitor the match between what they are expecting and what they are hearing. This is believed to be the cause of onset disfluencies (Howell, 2004; Malloy et al., 2022; Zimmermann et al., 1988) and syllabic serial exchanges in speech when DAF is introduced (Malloy et al., 2022). A singer might feel as though they cannot keep up with DAF.

Our findings therefore indicate that auditory imagery may benefit singers in maintaining their tonal reference and performing with consistent accuracy, as seen in previous studies (Brown & Palmer, 2012; Edmonson, 1972; Finney & Palmer, 2003; Goebl & Palmer, 2008; Highben & Palmer, 2003; MacRitchie & Milne, 2017). However, it remains unclear how imagery ability can be developed in the domain of music. Existing research using BAIS suggests that auditory imagery correlates only weakly with musical training. Reliance on external auditory feedback decreases with training, particularly classical vocal training (Bottalico et al., 2016, 2017). We found no correlation between BAIS score and years of studying music theory but did find a relationship between BAIS score and years of experience of performing. Partial correlation analyses showed that BAIS was associated with less per-participant TRD in pitch-shifted AAF, even when controlling for years of experience of performing. This was not the case for DAF (CV) effects, implying again that other factors may have influenced internal tempo references. We suggest that performing music in any context can be valuable in imagery training; formal study of music theory is not necessary. Although the environments in which people study music theory (e.g., university music departments) might provide opportunities to perform, we recruited several participants who maintain active professional and semi-professional musical careers despite having had no formal training; indeed, this is common among performing musicians. There remain two possibilities: (1) high BAIS-scoring individuals are more likely to become successful performers, or (2) experience of performing helps individuals develop auditory imagery ability. We suggest that musicians need to maintain access to and train their imagery if they are to thrive as performers. For example, the PETTLEP (Physical, Environment, Task, Timing, Learning, Emotion, Perspective) model in sports has been used to enable athletes to construct multi-modal images of their techniques during training (Wakefield et al., 2013). This model has also been shown to be beneficial for musicians’ performance (Wright et al., 2014) and could also be used for training auditory imagery, perhaps in the context of rehearsals in non-ideal conditions or with artificially introduced AAF.

### Limitations

Two considerations should be taken into account when interpreting these results and planning further studies. First, it is possible that confounding factors resulted from the use of participant-selected pieces such that accuracy was affected in an unknown way (SR-2). Different pieces may have had qualitatively different effects with AAF, such that some randomly toggled sections may have been aligned so as to be more in time, or the length of DAF on pitch overlaps may have created unexpected tonalities in some pieces but not others (Pfordresher & Palmer, 2002). However, we argue that we successfully negotiated the fundamental trade-off between undertaking a real-world task and controlling for every interaction. Although it is easier to analyze effects on the performance of isolated tasks such as tapping or pitch matching in the context of an experiment, we believe that our more ecologically valid approach was effective, not least because participants were not exposed to the stress potentially associated with sight singing. We therefore recommend the inclusion of participant-selected stimuli to future researchers.

Second, generalizations based on our results should be made with caution because our sample was small, given the constraints on recruitment and the intensive nature of the experimental task, and we therefore chose to conduct 2 × 3 × 4 ANOVAs, examining between-group effects and interactions of interest by using a median split to divide participants into two groups. Mixed-effects and multiple regression analyses, among others, may be more useful for identifying complex interactions in studies using larger samples. Although our approach to statistical analysis was acceptable since our chosen predictors were uncorrelated, we categorized participants, all of whom scored 4 or more on BAIS and therefore could be said to have reasonably good auditory imagery ability, dichotomously as high or low scorers (McClelland et al., 2015), thus potentially producing conservative results with small effect sizes (Iacobucci et al., 2015). Nevertheless, our results were significant, and we would therefore predict similar effects of BAIS score in future studies using different methods of analysis. We hope our work will be used as the basis for future studies with larger samples to confirm and validate our findings.

### Future work

To the best of our knowledge, no research has yet been conducted on temporal, as opposed to tonal, drift in unaccompanied singing. It would be worth exploring typical temporal drift in the light of the compensation for delay discussed in the speech, language, and hearing research literature. For instance, participants sang more slowly in DAF conditions (Pfordresher & Palmer, 2002), perhaps to compensate by trying to reduce the discrepancy between what they expected and what they were hearing. IOIs at binary subdivisions of the delay could be used to investigate this behavior and find out how long the delay can be before it becomes unmanageable.

The findings of the present study add to what is already known about the relationship between auditory and kinesthetic imagery by highlighting the use of internal and external methods of timekeeping and the sensory-based perception of movement on timing accuracy. Blended-imagery models, such as the MMIA, could be used to examine the temporal elements of auditory imagery.

In future research, the sound of the unaltered voice could be masked by other sources of sound (Parrell & Niziolek, 2021; Pfordresher & Mantell, 2012) when presenting AAF stimuli, although it would still be difficult to mask the sound produced by bone conduction. Just-noticeable-difference tasks could be used to contextualize our TRD and CV results by indicating the thresholds at which alterations in pitch, delay, and additional auditory stimuli are perceptible.

## Conclusion

We examined participants’ ability to maintain tonal and temporal accuracy while singing familiar music under combinations of AAF conditions and forced audiation and voice distraction tasks. Participants with greater auditory imagery ability, as measured using BAIS, produced more consistent tonal accuracy with pitch-shifted feedback and in forced audiation tasks, particularly with whole-tone pitch-shifts. We found no significant interaction between BAIS score and temporal accuracy; DAF significantly affected both high- and low-BAIS participants, but this resulted in less temporal deviation than when they performed in control conditions. These results support multimodal imagery theories: the ability to maintain temporal reference and timing consistency is likely not dependent on auditory imagery alone but is also dependent on other factors such as kinesthetic imagery and the prioritization of accuracy over expression. Finally, we found that auditory imagery ability correlated with more years of experience of performing, rather than years of studying music theory formally. This may be the result of learning through performing to adapt to non-ideal feedback and suggests that auditory imagery could be trained through the practice of performing.

## Supplemental Material

sj-pdf-1-msx-10.1177_10298649231223077 – Supplemental material for Auditory imagery ability influences accuracy when singing with altered auditory feedback Supplemental material, sj-pdf-1-msx-10.1177_10298649231223077 for Auditory imagery ability influences accuracy when singing with altered auditory feedback by Courtney N. Reed, Marcus Pearce and Andrew McPherson in Musicae Scientiae

## Notes

1. Some participants did not complete all task-condition combinations due to external constraints. See Supplemental Table 3 for details.
