# Supplementary material for: Auditory imagery ability influences accuracy when singing with altered auditory feedback
Source: Music Sci. 2024 Feb 15;28(3):478–501. doi: 10.1177/10298649231223077 (PMC11357896; doi:10.1177/10298649231223077)
Supplement: sj-pdf-1-msx-10.1177_10298649231223077 – Supplemental material for Auditory imagery ability influences accuracy when singing with altered auditory feedback [file sj-pdf-1-msx-10.1177_10298649231223077.pdf]

Supplemental Tables

Supplemental Table 1: Participant Demographics & Experience

ID	Principal Instrument	Other Instruments	Nationality	BAIS-V	BAIS-C	Performance Experience (years)	Music Theory Study (years)	Formal Training (Y/N)
1	Piano	-	Taiwan	4.64	5	8	10	Yes
2	Guitar	-	Ireland, USA	5	4.86	10	10	Yes
3	Flute	-	UK	6.14	6.14	16	10	Yes
4	Voice	-	UK	4.79	5.5	18	11	Yes
5	Voice	Violin, Guitar	UK	5.21	4.64	3	3	Yes
6	Voice	-	UK	4.86	4.71	2	0.5	No
7	Voice	Recorder, Piano, Clarinet	Portugal	5.79	5.71	20	4	Yes
8	Piano	-	China	5.07	3.79	12	2	Yes
9	Sampler/EDI*	-	UK, Australia	5.93	6	24	1	No
10	Voice	-	UK, USA	4.36	3.79	1	0.5	No
11	Voice	Guitar, Ukulele, Piano	UK	4.86	5.36	11	7	Yes
12	Voice	-	Costa Rica	5.57	5.79	11	7	No
13	Voice	-	India	5.64	6.21	9	20	Yes
14	Dhol	-	India	6.57	6.43	12	6	Yes
15	Voice	Violin, Piano	UK	4.5	4.86	7	3	Yes
16	Guitar	-	Italy	4.14	4.86	10	10	Yes

Supplemental Table 1. Participant information including principal instrument, demographics, performance experience, and musical training provided alongside respective scores on the BAIS-V and BAIS-C subscales (*Electronic Digitial Instrument).

Supplemental Table 2: Participant-Chosen Pieces

Supplemental Table 2 follows on the subsequent page.

ID	Song (Artist/Soundtrack)	Key	Tempo (bpm)	Meter	Beat Length (ms)	Bar Length (ms)	MIDI toolbox Complexity Measures				
							ambitus	nPVI	notedensity	tonalstability	complebm
1	Bizarre Love Triangle (Frente!)	Db maj	250	8/8	240	1920	12	39.117	1.1938	4.392	4.7504
2	New Slang (The Shins)	C maj	130	4/4	462.54	1846.15	16	25.113	0.96104	3.1655	3.7666
3	Where No One Stands Alone (The Peasall Sisters)	Bb maj	70	3/4	857.14	2571.43	17	67.21	0.80288	3.9503	2.7102
4	There's a Fine Fine Line (from the musical *Avenue Q*)	G maj	60	4/4	1000	4000	19	38.486	0.95789	3.8457	3.8514
5	Mr. Snow (from the musical *Carousel*)	G maj	65	2/2	923.08	1846.15	17	36.571	0.92545	3.7231	3.6752
6	Tears in the Typing Pool (Broadcast)	A min	80	9/8	750	6750	15	37.887	0.92364	3.8757	3.6609
7	Fantoches (*Fêtes Galantes*, Debussy)	C maj	110	2/4	545.45	1090.91	19	40.137	1.272	4.0661	4.1736
8	City of Stars (Mia's Solo) (from the film *La La Land*)	F maj	120	4/4	500	2000	20	50.939	0.72189	4.7393	3.7685
9	We Only Come Out At Night (Smashing Pumpkins)	C maj	60	4/4	1000	4000	19	43.81	0.84679	4.1044	3.5586
10	American Pie (Don McLean)	E min	140	4/4	428.57	1714.29	24	42.181	1.1435	4.2247	3.9507
11	Voi Che Sapete (*Le nozze di Figaro*, Mozart)	Bb maj	65	2/4	800	1600	17	40.844	1.5809	4.6399	4.9772
12	Proud Mary (Creedence Clearwater Revival)	D maj	60	4/4	1000	4000	14	35.367	1.0896	3.7356	3.5572
13	Bharat (from the film *Manikarnika*)	D maj	60	4/4	1000	4000	15	33.401	1.1255	3.4381	4.3185
14	My Heart Will Go On (Celene Dion)	E maj	60	4/4	1000	4000	14	68.401	0.75	4.1741	3.6365
15	Agnus Dei (*Krönungsmesse*, Mozart)	F maj	60	3/4	1000	4000	17	51.99	0.85535	3.7956	4.2806
16	Back Pocket (Vulfpeck)	D maj	90	4/4	666.67	2666.69	13	29.955	1.8895	4.5944	4.3794

Supplemental Table 2. Participant-chosen songs as performed with the reference tempo and key centre agreed at the start of the trials. Reference tempo and the first two bars for tonal reference were provided at the start of each trial. Timing factors and complexity measures are presented for each piece (NB: *ambitus* = melodic range (semitones), *complebm* = melodic complexity, *nPVI* = durational variability of note events).

Supplemental Table 3: Performance Combinations

		Condition					
		Normal Feedback	Headphone Feedback	200 ms Delay	600 ms Delay	+ 1/4 Tone Pitch Shift	+ Whole Tone Pitch Shift
Task	Normal	16	16	16	16	11	16
	Toggled	16	16	16	16	11	16
	Toggled & Voice Distraction	14	14	14	14	9	13

Supplemental Table 3. Performances included for each task-condition combination: The 1/4 Tone Pitch Shift condition was introduced after the initial five participants. Participants 6 and 13 did not complete the Toggled & Voice Distraction tasks and Participant 5 was not able to complete the performance in the Whole Tone Pitch Shift condition in the Toggled & Voice Distraction task due to time constraints.

Supplemental Table 4: Participant BAIS Grouping

ID	Aggregate BAIS	BAIS Group	Performance Experience (years)	Music Theory Study (years)
10	4.075	Low	1	0.5
8	4.43	Low	12	2
16	4.5	Low	10	10
15	4.68	Low	7	3
6	4.785	Low	2	0.5
1	4.82	Low	8	10
5	4.925	Low	3	3
2	4.93	Low	10	10
11	5.11	High	11	7
4	5.145	High	18	11
12	5.68	High	11	7
7	5.75	High	20	4
13	5.925	High	9	20
9	5.965	High	24	1
3	6.14	High	16	10
14	6.5	High	12	6

Supplemental Table 4. Participant Demographics: Participants are ordered by aggregate BAIS score, demonstrating the median split and the relation of other demographic information.

Supplemental Results (SR)

SR-1: Examining Groups by BAIS Score, Formal Training, and Primary Instrument

Preliminary analysis was conducted to establish any group differences in terms of musical imagery ability as measured by BAIS-V and BAIS-C scores. The groups were divided based on indications made at the time of sign-up and again on the Gold-MSI of (1) being primarily a vocalist or an instrumentalist and (2) having been formally trained on the principal instrument or not.

Vocalists vs. Instrumentalists. A two-sample t-test was performed to determine whether the groups differed significantly in either BAIS subscale. There was no significant difference in the BAIS-V scores of the vocalists (M = 5.18, SD = 0.81) and the instrumentalists (M = 5.2, SD = 0.56), t(14) = – 0.06, p = .95. This was also true of BAIS-C scores between the vocalists (M = 5.23, SD = 0.99) and the instrumentalists (M = 5.22, SD = 0.57), t(14) = 0.01, p = .99).

Formal Training. For BAIS-V score, there was no significant difference between the participants with formal training (M = 5.26, SD = 0.7) and those without (M = 5.0, SD = 0.69), t(14) = 0.63, p = .54). This was also the case for BAIS-C, t(14) = 1.38, p = .24, although variance between the participants with formal training (M = 5.42, SD = 0.64) and those without (M = 4.64, SD = 1.07) was unequal. Given the lack of any statistical differences in BAIS scores between vocalists and instrumentalists, or participants with and without formal training, no further distinction was made between participants on these grounds. All participants were examined as a single cohort of confident, skilled singers regularly performing vocals in some capacity.

Aggregating BAIS Scores. Additionally, participants' BAIS scores from the two subscales had a strong positive correlation (r = .76, R2 = .57, $p < .001$, Figure SR-1.1). This is consistent with previous research using the questionnaire (Halpern 2015; Pfordresher and Halpern 2013) and indicates that individuals with ability to produce a more vivid auditory image may also have greater control over that image. Given this strong relationship between the BAIS subscales, BAIS-V and BAIS-C scores were averaged for each participant and this aggregate BAIS score (referred to simply as "BAIS score") is used in all further analyses.

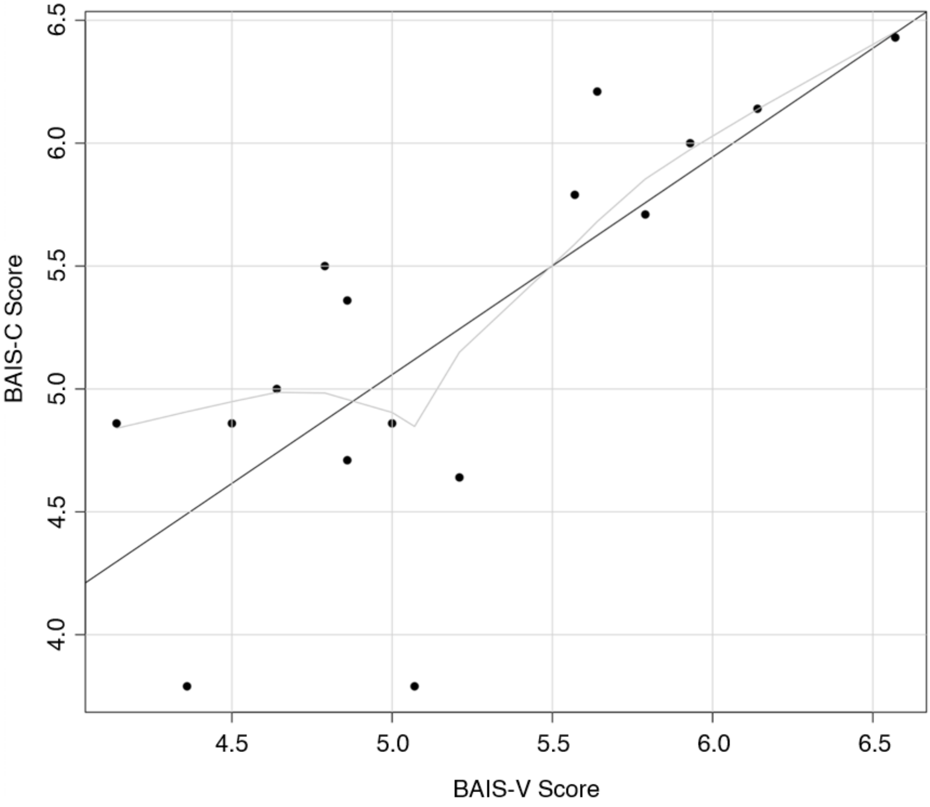

Figure SR- 1.1. Positive correlation between participant scores on the BAIS subscales linear (black) and loess (grey) regression.

SR-2: Examining Potential Covariates in Self-Selected Music

We also determined whether the participants' self-selected pieces might introduce covariates into the analyses.

Music Complexity. Several elements of complexity were used to compare the different participant-chosen pieces; these measures were calculated using the MIDI toolbox functions for MATLAB (Eerola and Toiviainen 2004) and included the melodic range in semitones (ambitus), melodic complexity (complebm, derived from the expectancy-based model of melodic complexity by Eerola and North (2000) and Schaffrath (1995)), durational variability of note events (nPVI, by Grabe and Low (2002) and Patel and Daniele (2003)), note density as number of notes per beat (notedensity), and tonal stability of notes in a melody (tonality, by Krumhansl (1990)).

Correlations between these features in each song and the respective participant's BAIS score, years of study, and years of performance experience were all found to be weak (r $< \pm$ 0.39). Ambitus and years of study (r = -0.48), note density and melodic complexity (r = .66), BAIS score and years of performance (r = .6), and durational variability of note events with note density (r = -.52), melodic complexity (r = -0.44), and BAIS score (r = 0.43) were found to be moderately correlated (Figure SR-2.1). However, these were all found to be non-significant (p $>$ .05). As the complexity of the pieces in these dimensions did not correlate with any of our participant measures, we assume that the participant-selected music does introduce confounding factors in our primary analyses.

Interaction with Delays. Additionally, potential interactions with DAF were examined; given previous research on timing accuracy with delays (Pfordresher and Palmer 2002), it is possible that the event timing of some of the chosen pieces might result in DAF occurring at a binary subdivision of the beat, thus creating a less distracting delay for some participants. For instance, if the IOI between the notable beats of the measure was 400 ms, the 200 ms DAF condition would result in delays occurring at a subdivision of the beat and could potentially benefit a participant's accuracy in timekeeping. In order to determine whether this was an applicable factor in any of the participant-selected pieces, the IOI between notable beats in each piece was determined using the reference tempo in BPM (provided at the start of each performance) and the meter of the piece. The IOI of notable beats, as well as the measure length, in milliseconds is presented in Supplemental Table 2.

In both cases, the length of the beat and the measure for all pieces would not provide reasonable binary subdivisions with the two DAF conditions; we assume interactions with delays are a likely confounding factor in comparison of participant experience and performance outcomes in the DAF conditions.

SR-3: Control Testing between NF and HF Tasks

As all AAF conditions were delivered via headphones, it was first necessary to test whether using headphones had significant impact on accuracy compared to singing without headphones. For each of the three accuracy measures, the non-AAF performances done in the Normal Feedback (NF) control conditions were compared with

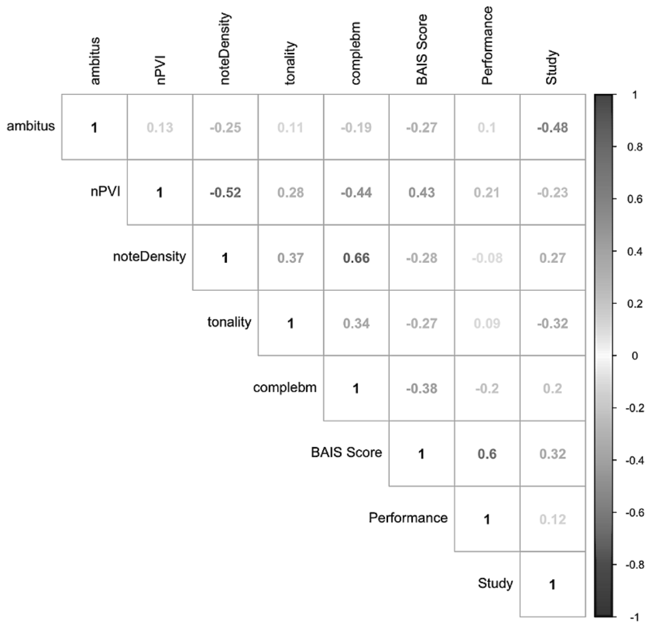

Figure SR- 2.1. Correlation matrix between participant experience measures (BAIS score, years of performance experience, and years of theory study) and respective complexity measures for each piece calculated with MIDI toolbox functions (NB: *ambitus* = melodic range (semitones), *complebm* = melodic complexity, *nPVI* = durational variability of note events).

the Headphones Feedback (HF) performances in a Welch Two-Sample t-test. There was no statistical difference between these performances for any of the accuracy measures: TRD, $t(82) = 0.32$, $p = .75$; CV, $t(86) = 0.27$, $p = .79$; MBs, $t(54) = 0.34$, $p = .73$. Given there appears to be no effect of headphones within the feedback loop, we use HF performances as controls for further analyses and assume their use does not confound responses to AAF stimuli.

To ensure that BAIS score would not be a confounding factor within this control performance (e.g., confident singers should be able to perform well without AAF, regardless of imagery skill) and to justify using an average control score for the group in group-adjusted analyses, a correlation analysis was performed. There were no significant or strong correlations found between participants' aggregate BAIS scores and their controls. For TRD in the Normal task, $r = 0.28$, $p = .29$; Toggled, $r = 0.038$, $p = .89$; TVD $r = -0.17$, $p = .56$. For CV, Normal task, $r = 0.28$, $p = .29$; Toggled, $r = 0.34$, $p = .2$; TVD, $r = 0.11$, $p = .72$). For MBs, Toggled, $r = 0.31$, $p = .24$; TVD: $r = -0.053$, $p = .86$). We therefore assume control performance accuracy itself is not affected by BAIS score.

SR-4: Additional Details for Individual-Adjusted TRD Analysis

TRD scores were transformed with Ordered Quantile Normalizing Transformation (ORQ) (Peterson and Cavanaugh 2019). There was no significant three-way interaction between the independent variables; a planned simple two-way fit for each BAIS group showed the effect of condition on TRD was significant for the low-BAIS group, $F(3,144) = 3.70$, $p = .013$. Bonferroni-adjusted pairwise comparisons show a significant difference between the two groups, $t(162) = -2.97$, $p < .001$, in the Whole Tone Pitch Shift condition. Low-BAIS participants had higher TRD compared to their control performances than high-BAIS participants. The Toggled & Voice Distraction task differed significantly between the groups, $t(160) = 3.69$, $p < .001$, with low BAIS participants again having higher TRD compared to their control performances. Full-factorial results for individual-adjusted TRD are found in Table SR-4.1.

Effect	DFn	F	p
Task	2	0.672	0.512
Condition	3	1.154	0.330
BAIS Group	1	3.799	0.053
Task : Condition	6	0.796	0.575
Task : BAIS Group	2	3.304	**0.040** *
Condition : BAIS Group	3	3.628	**0.015** *
Task : Condition : BAIS Group	6	1.481	0.189

(a) 2x3x4 Analysis of Variance (ANOVA Type II)

BAIS Group	Effect	DFn	F	p
High	Condition	3	1.28	0.283
High	Task	2	2.9	0.058
High	Condition : Task	6	1.27	0.277
Low	Condition	3	3.70	**0.013** *
Low	Task	2	1.03	0.359
Low	Condition : Task	6	1.01	0.421

(b) Two-Way Interaction by Group (ANOVA Type II)

Condition	Statistic	p
Delay 200 ms	-0.646	0.519
Delay 600 ms	0.721	0.472
¼ Pitch Shift	-0.145	0.885
Whole Tone Pitch Shift	-3.69	**0.000305** *

(c) Group Pairwise Comparisons (Bonferroni-adjusted), Condition

Task	Statistic	p
Toggle	-0.395	0.694
Toggled & Voice Dist.	-2.97	**0.00345** *

(d) Group Pairwise Comparisons (Bonferroni-adjusted), Task

Condition A	Condition B	Estimate	p
Delay 200 ms	Delay 600 ms	-0.127	0.984
Delay 200 ms	¼ Pitch Shift	-0.276	0.872
Delay 200 ms	Whole Tone Pitch Shift	0.660	0.261
Delay 600 ms	Whole Tone Pitch Shift	-0.149	0.976
Delay 600 ms	¼ Pitch Shift	0.786	0.139
¼ Pitch Shift	Whole Tone Pitch Shift	0.935	0.0712

(e) Tukey's HSD, Low BAIS Group, Condition

Task A	Task B	Estimate	p
Toggled	Toggled & Voice Dist.	-0.229	0.902

(f) Tukey's HSD, Low BAIS, Task

Table SR- 4.1. Full-factorial results from analysis of the effect on individual-adjusted TRD by interaction between BAIS Group, Condition, and Task.

SR-5: Additional Details for Individual-Adjusted CV Analysis

Individual-adjusted CV scores were normally distributed. Planned two-way analyses between each BAIS group showed significant effect of condition on CV for the low-BAIS group, $F(3,144) = 8.22$, $p < .001$. Bonferroni-adjusted pairwise comparisons indicated significant differences between groups for both the 200 ms DAF, $t(160) = 2.85$, $p = .005$, and the 600 ms DAF, $t(160) = 2.34$, $p = .021$. Full-factorial results for individual-adjusted CV are found in Table SR-5.1.

Effect	DFn	F	p
Task	2	0.233	0.792
Condition	3	7.321	**0.000133** *
BAIS Group	1	7.323	**0.008** *
Task : Condition	6	0.683	0.664
Task : BAIS Group	2	0.179	0.836
Condition : BAIS Group	3	0.657	0.179
Task : Condition : BAIS Group	6	0.457	0.839

(a) 2x3x4 Analysis of Variance (ANOVA Type II)

BAIS Group	Effect	DFn	F	p
High	Condition	3	0.884	0.451
High	Task	2	0.521	0.959
High	Condition : Task	6	0.451	0.842
Low	Condition	3	8.22	**0.0000438** *
Low	Task	2	0.002	0.998
Low	Condition : Task	6	0.688	0.659

(b) Two-Way Interaction by Group (ANOVA Type II)

Condition	Statistic	p
Delay 200 ms	2.85	**0.00495** *
Delay 600 ms	2.34	**0.0205** *
¼ Pitch Shift	0.243	0.808
Whole Tone Pitch Shift	-0.0108	0.991

(c) Group Pairwise Comparisons (Bonferroni-adjusted), Condition

Condition A	Condition B	Estimate	p
Delay 200 ms	Delay 600 ms	0.512	0.962
Delay 200 ms	¼ Pitch Shift	3.21	**0.0325** *
Delay 200 ms	Whole Tone Pitch Shift	3.29	**0.021** *
Delay 600 ms	Whole Tone Pitch Shift	2.69	0.0894
Delay 600 ms	¼ Pitch Shift	2.78	0.0625
¼ Pitch Shift	Whole Tone Pitch Shift	0.0899	0.99

(d) Tukey's HSD, Low BAIS Group, Condition

Table SR- 5.1. Full-factorial results from analysis of the effect on individual-adjusted CV by interaction between BAIS Group, Condition, and Task.

SR-6: Additional Details for Individual-Adjusted MBs Analysis

Full-factorial results for individual-adjusted MBs are found in Table SR-6.1.

Effect	DFn	F	p
Task	1	2.646	0.107
Condition	3	2.615	0.056
BAIS Group	1	0.319	0.574
Task : Condition	3	0.977	0.407
Task : BAIS Group	1	0.051	0.822
Condition : BAIS Group	3	0.363	0.780
Task : Condition : BAIS Group	3	0.261	0.853

(a) 2x3x4 Analysis of Variance (ANOVA Type II)

BAIS Group	Effect	DFn	F	p
High	Condition	3	1.75	0.162
High	Task	1	1.57	0.214
High	Condition : Task	3	0.902	0.443
Low	Condition	3	1.26	0.293
Low	Task	1	1.10	0.298
Low	Condition : Task	3	0.336	0.8

(b) Two-Way Interaction by Group (ANOVA Type II)

Table SR- 6.1. Full-factorial results from analysis of the effect on individual-adjusted MBs by interaction between BAIS Group, Condition, and Task.

SR-7: Additional Details for Group-Adjusted TRD Analysis

A planned two-way analysis by BAIS group showed a significant effect of condition on TRD for the low BAIS group, $F(3,144) = 2.78$, $p = .043$. Bonferroni-adjusted pairwise comparisons between the BAIS groups revealed a significant difference in the Whole Tone Pitch Shift condition only, $t(160) = -2.00$, $p = .047$, with low-BAIS scorers having higher TRD scores than the group average. Full-factorial results for the group-adjusted TRD can be found in Table SR-7.1.

Effect	DFn	F	p
Task	2	0.355	0.702
Condition	3	1.223	0.304
BAIS Group	1	0.010	0.921
Task : Condition	6	0.470	0.830
Task : BAIS Group	2	0.549	0.579
Condition : BAIS Group	3	1.815	0.147
Task : Condition : BAIS Group	6	1.081	0.377

(a) 2x3x4 Analysis of Variance (ANOVA Type II)

BAIS Group	Effect	DFn	F	p
High	Condition	3	0.367	0.777
High	Task	2	0.115	0.891
High	Condition : Task	6	0.817	0.558
Low	Condition	3	2.78	**0.043** *
Low	Task	2	0.757	0.471
Low	Condition : Task	6	0.734	0.623

(b) Two-Way Interaction by Group (ANOVA Type II)

Condition	Statistic	p
Delay 200 ms	0.00421	0.997
Delay 600 ms	1.125	0.213
¼ Pitch Shift	0.613	0.541
Whole Tone Pitch Shift	-2.00	**0.047** *

(c) Group Pairwise Comparisons (Bonferroni-adjusted), Condition

Condition A	Condition B	Estimate	p
Delay 200 ms	Delay 600 ms	-0.118	0.996
Delay 200 ms	¼ Pitch Shift	-0.261	0.961
Delay 200 ms	Whole Tone Pitch Shift	0.578	0.679
Delay 600 ms	Whole Tone Pitch Shift	-0.143	0.993
Delay 600 ms	¼ Pitch Shift	0.696	0.54
¼ Pitch Shift	Whole Tone Pitch Shift	0.839	0.41

(d) Tukey's HSD, Low BAIS Group, Condition

Table SR- 7.1. Full-factorial results from analysis of the effect on group-adjusted TRD by interaction between BAIS Group, Condition, and Task.

SR-8: Additional Details for Group-Adjusted CV Analysis

Further two-way comparisons by BAIS group revealed a significant effect of condition on CV for both the high- (F[3,144] = 3.36, p = .02) and low-BAIS groups (F[3,144]) = 10.6, p < .0001). Full-factorial results for the group-adjusted CV can be found in Table SR-8.1.

Effect	DFn	F	p
Task	2	0.262	0.77
Condition	3	12.721	**2.01e-07 ***
BAIS Group	1	0.017	0.91
Task : Condition	6	0.513	0.79
Task : BAIS Group	2	0.225	0.79
Condition : BAIS Group	3	1.220	0.31
Task : Condition : BAIS Group	6	0.394	0.88

(a) 2x3x4 Analysis of Variance (ANOVA Type II)

BAIS Group	Effect	DFn	F	p
High	Condition	3	3.36	**0.02 ***
High	Task	2	0.574	0.565
High	Condition : Task	6	0.22	0.97
Low	Condition	3	10.6	**2.37e-6 ***
Low	Task	2	0.0007	0.993
Low	Condition : Task	6	0.687	0.66

(b) Two-Way Interaction by Group (ANOVA Type II)

Condition	Statistic	p
Delay 200 ms	0.995	0.321
Delay 600 ms	0.633	0.527
¼ Pitch Shift	-0.208	0.835
Whole Tone Pitch Shift	-1.64	0.104

(c) Group Pairwise Comparisons (Bonferroni-adjusted), Condition

Table SR- 8.1. Full-factorial results from analysis of the effect on group-adjusted CV by interaction between BAIS Group, Condition, and Task.

SR-9: Additional Details for Group-Adjusted MBs Analysis

Two-way interaction by group revealed a significant effect of task for the low-BAIS group only, F(1,93) = 5.57, p = .02. Full-factorial results for the group-adjusted MBs can be found in Table SR-9.1.

Effect	DFn	F	p
Task	1	3.442	0.067
Condition	3	2.421	0.071
BAIS Group	1	0.106	0.745
Task : Condition	3	0.883	0.453
Task : BAIS Group	1	2.000	0.161
Condition : BAIS Group	3	0.567	0.638
Task : Condition : BAIS Group	3	0.155	0.926

(a) 2x3x4 Analysis of Variance (ANOVA Type II)

BAIS Group	Effect	DFn	F	p
High	Condition	3	2.09	0.107
High	Task	1	0.081	0.777
High	Condition : Task	3	0.733	0.535
Low	Condition	3	0.94	0.425
Low	Task	1	5.57	**0.02 ***
Low	Condition : Task	3	0.305	0.821

(b) Two-Way Interaction by Group (ANOVA Type II)

Task	Statistic	p
Toggle	-0.629	0.531
Toggled & Voice Dist.	1.46	0.146

(c) Group Pairwise Comparisons (Bonferroni-adjusted), Task

Table SR- 9.1. Full-factorial results from analysis of the effect on group-adjusted MBs by interaction between BAIS Group, Condition, and Task.

References

Eerola, T. and North, A. C. (2000). Expectancy-Based Model of Melodic Complexity. In Woods, C., Luck, G. B., Brochard, R., O'Neill, S. A., and Sloboda, J. A., editors, *Proc. Sixth International Conference on Music Perception and Cognition (ICMPC). Keele, Staffordshire, UK*, pages 1–7.

Eerola, T. and Toiviainen, P. (2004). *MIDI toolbox: MATLAB tools for music research*. Kopijyvä, Jyväskylä, Finland, University of Jyväskylä.

Grabe, E. and Low, E. L. (2002). Durational variability in speech and the Rhythm Class Hypothesis. In *Laboratory Phonology 7*, pages 515–546. De Gruyter Mouton.

Halpern, A. R. (2015). Differences in auditory imagery self-report predict neural and behavioral outcomes. *Psychomusicology: Music, Mind and Brain*, 25:37.

Krumhansl, C. L. (1990). *Cognitive Foundations of Musical Pitch*. Oxford University Press, New York.

Patel, A. D. and Daniele, J. R. (2003). An empirical comparison of rhythm in language and music. *Cognition*, 87:B35–B45.

Peterson, R. A. and Cavanaugh, J. E. (2019). Ordered quantile normalization: a semiparametric transformation built for the cross-validation era. *Journal of Applied Statistics*, 47(13-15):2312–2327.

Pfordresher, P. Q. and Halpern, A. R. (2013). Auditory imagery and the poor-pitch singer. *Psychonomic Bulletin and Review*, 20:747–753.

Pfordresher, P. Q. and Palmer, C. (2002). Effects of delayed auditory feedback on timing of music performance. *Psychological Research*, 16:71–79.

Schaffrath, H. (1995). *The Essen folksong collection in kern format. [computer database]*. Center for Computer Assisted Research in the Humanities, Menlo Park, CA.
